# Predictors of Subclinical Cardiovascular Disease in Women with Polycystic Ovary Syndrome: Interrelationship of Dyslipidemia and Arterial Blood Pressure

**DOI:** 10.1155/2015/812610

**Published:** 2015-03-24

**Authors:** Djuro Macut, Marina Bačević, Ivana Božić-Antić, Jelica Bjekić-Macut, Milorad Čivčić, Snježana Erceg, Danijela Vojnović Milutinović, Olivera Stanojlović, Zoran Andrić, Biljana Kastratović-Kotlica, Tijana Šukilović

**Affiliations:** ^1^Clinic of Endocrinology, Diabetes and Diseases of Metabolism, Faculty of Medicine, University of Belgrade, 11000 Belgrade, Serbia; ^2^General Hospital Pančevo, 26000 Pančevo, Serbia; ^3^University Medical Center “Bežanijska Kosa”, 11080 Belgrade, Serbia; ^4^Department of Biochemistry, Institute for Biological Research “Siniša Stanković”, University of Belgrade, 11060 Belgrade, Serbia; ^5^Institute of Medical Physiology, Faculty of Medicine, University of Belgrade, 11000 Belgrade, Serbia; ^6^Clinic of Obstetrics and Gynecology, Faculty of Medicine, University of Belgrade, 11000 Belgrade, Serbia; ^7^Faculty of Mathematics, University of Belgrade, 11000 Belgrade, Serbia

## Abstract

*Background*. Women with polycystic ovary syndrome (PCOS) could develop subclinical atherosclerosis during life.* Purpose*. To analyze cardiovascular risk (CVR) factors and their relation to clinical markers of cardiovascular disease (CVD) in respect to their age.* Material and Methods*. One hundred women with PCOS (26.32 ± 5.26 years, BMI: 24.98 ± 6.38 kg/m^2^) were compared to 50 respective controls. In all subjects, total cholesterol (TC), HDL-C, LDL-C, triglycerides, TC/HDL-C and TG/HDL-C ratios, glucose, insulin and HOMA index, waist-to-hip ratio (WHR), systolic and diastolic blood pressure (SBP and DBP, resp.), and carotid intima-media thickness (CIMT) were analyzed in respect to their age and level of androgens.* Results*. PCOS over 30 years had higher WHR (*P* = 0.008), SBP (*P* < 0.001), DBP (*P* < 0.001), TC (*P* = 0.028), HDL-C (*P* = 0.028), LDL-C (*P* = 0.045), triglycerides (*P* < 0.001), TC/HDL-C (*P* < 0.001), and triglycerides/HDL-C (*P* < 0.001) and had more prevalent hypertension and pronounced CIMT on common carotid arteries even after adjustment for BMI (*P* = 0.005 and 0.036, resp.). TC/HDL-C and TG/HDL-C were higher in PCOS with the highest quintile of FAI in comparison to those with lower FAI (*P* = 0.045 and 0.034, resp.).* Conclusions*. PCOS women older than 30 years irrespective of BMI have the potential for early atherosclerosis mirrored through the elevated lipids/lipid ratios and through changes in blood pressure.

## 1. Introduction

Polycystic ovary syndrome (PCOS) is the most common endocrine disorder in premenopausal women, with a prevalence of 6–10%, and it is characterized by hyperandrogenic features and chronic oligoanovulation [1, 2]. In addition to obesity, insulin resistance and an impaired *β* cell function of the pancreas, dyslipidemia, and hypertension are more prevalent compared to the general population. An altered lipid profile in PCOS women is characterized by elevated triglycerides, normal or increased concentrations of total and LDL cholesterol, decreased HDL cholesterol, and an increase in specific lipid ratios [3, 4].

Several studies have shown that women with PCOS have an increased prevalence of hypertension. While some authors showed that a higher prevalence of hypertension was related to the obesity and not to PCOS* per se* [5], others demonstrated that a high value of the free androgen index (FAI) in even young women with PCOS was associated with hypertension independent of insulin resistance, body mass index (BMI), and dyslipidemia [6]. Controversy over the cardiovascular disease (CVD) and outcomes in women with PCOS continues towards menopause. Shaw et al. have shown that menopausal women with PCOS characteristics more frequently had diabetes, obesity, metabolic syndrome (MetS), and signs of coronary heart disease (CHD) [7]. However, other authors did not find an association between PCOS and CHD or increased rates of cardiovascular morbidity and mortality [8].

Endothelial dysfunction is one of the earliest signs of subclinical atherosclerosis. If we consider that women with PCOS are exposed to risk factors for CVD early in life, the diagnosis of subclinical atherosclerosis in this population would be of importance. Ultrasonographic measurement of the carotid intima-media thickness (CIMT) is a reliable and noninvasive method for the determining of preclinical atherosclerosis. Increased CIMT is correlated with an increased risk of cardiovascular events such as myocardial infarction and stroke in the general population [9].

Taking into consideration the probability of the existence of preclinical CVD in women with PCOS, the aim of this study was to analyse the relation between lipid disarrangement, as the most frequent metabolic abnormality in PCOS, and markers of CVD, primarily arterial blood pressure, in respect to their age and androgen levels.

## 2. Materials and Methods

### 2.1. Selection of the Subjects

The study included 100 women with a confirmed diagnosis of PCOS (mean age 26.32 ± 5.26 years and BMI 24.98 ± 6.38 kg/m^2^) and 50 healthy controls (mean age 27.96 ± 5.66 years and BMI 24.66 ± 6.74 kg/m^2^). Subjects included in the study were referred to the outpatient endocrinology clinic, because of irregular menstrual cycles, hirsutism, or acne. PCOS was defined on the basis of the revised Rotterdam consensus in 2003 [10] which implies the presence of at least two of the following three criteria: moderate oligo/amenorrhea (fewer than eight menstrual cycles in a year), presence of clinical hyperandrogenism (hirsutism) and/or biochemical hyperandrogenemia [11], and ultrasound confirmation of the existence of polycystic ovaries. Hirsutism was defined as Ferriman-Gallwey score ≥8 [12]. Biochemical hyperandrogenemia was defined as total testosterone >2.0 nmol/L and/or FAI ≥6. Women with PCOS were analyzed during the early follicular phase of the regular menstrual cycle, or at any time if they had severe oligomenorrhea or were amenorrheic.

A control group of healthy women had no signs of hyperandrogenism. Normal ovulatory status in controls was confirmed by the measurement of serum progesterone levels during the luteal phase of the menstrual cycle. Progesterone >10 nmol/L was considered ovulatory. Normal appearance of the ovaries was confirmed by ultrasound examination. Woman with the presence of elevated fasting glucose (fasting venous glucose ≥6 mmol/L), pregnancy, history of alcohol or drug abuse, and medical history of breast cancer and uterine and cardiovascular disorders were excluded from the study. Also, hypothyroidism, nonclassical form of 21-hydroxylase deficit, hyperprolactinemia, Cushing's disease, and androgen-secreting tumors were excluded by appropriate tests. None of the subjects received oral contraceptives, glucocorticoids, antiandrogens, and other hormonal agents known to affect menstrual cyclicity or the drugs for the treatment of diabetes and obesity, for at least three months before the commencement of the study.

The study was approved by the Institutional Ethics Committee and all examined women gave written informed consent before the study.

### 2.2. Anthropometric and Biochemical Characteristics of the Subjects

In all subjects, BMI, waist-to-hip ratio (WHR), and systolic and diastolic arterial blood pressure (SBP and DBP, resp.) were determined. BMI (kg/m^2^) was calculated as the ratio of body weight (kg) and body height (m) squared. WHR was calculated as the ratio of the smallest waist circumference (cm) at the level of umbilicus to maximal hip circumference (cm). SBP (mmHg) and DBP (mmHg) were measured using mercury sphygmomanometer on the left arm in a sitting position by the same examiner. The average of two measurements was calculated. According to the criteria used in all definitions for metabolic syndrome [13], hypertension was defined as SBP ≥130 mmHg and/or DBP ≥85 mmHg. Determination of CIMT was performed by ultrasound, using linear probe (7.5–10 MHz) as previously described [14]. Measurement was performed on both common (ACC) and internal carotid arteries (ACI), and mean values of CIMT on ACC and ACI were determined.

Baseline blood samples were collected after 12 hours of fasting in the follicular phase of the cycle (between the 3rd and the 7th days) or randomly in a case of amenorrhea, and the following analyses were determined: total cholesterol (TC), HDL-cholesterol (HDL-C), LDL-cholesterol (LDL-C), triglycerides (TG), apolipoprotein A1 (Apo-A1), apolipoprotein B (Apo-B), glucose, insulin, total testosterone, sex-hormone-binding globulin (SHBG), androstenedione, and dehydroepiandrosterone sulfate (DHEAS). The samples for hormone analysis were stored on −80°C until measurement. TC (mmol/L) and TG (mmol/L) were measured by standard enzymatic methods (TC: cholesterol oxidase, Randox, Belfast, UK; TG: glycerol-3-phosphate oxidase, Randox, UK). HDL-C (mmol/L) was measured by the direct method (Randox, UK). LDL-C l (mmol/L) was determined by Friedewald formula. Apo-A1 (g/L) and Apo-B (g/L) were determined by nephelometric method (Behring, Marburg, Germany). Blood glucose (mmol/L) was measured by glucose oxidase method (Randox, UK), using the autoanalyzer (Beckman, Austria), and plasma insulin (mU/L) was measured using radioimmunoassay RIA INSULIN (PEG, INEP, Belgrade, Serbia). Serum total testosterone (nmol/L), androstenedione (ng/mL), DHEAS (nmol/L), and SHBG (nmol/L) were measured by radioimmunoassay (Testo-CT2, R-GM-100, DHEAS-CT, and SHBG-RIACT, resp.; CIS Bio International, Gifsur-Yvette, France). FAI was calculated by the formula [(100 × total testosterone)/SHBG]. Insulin resistance (IR) was determined using HOMA (homeostasis model assessment) index, which is calculated by the formula: [fasting insulin (mU/L) × fasting glucose (mmol/L)]/22.5. TC/HDL-C <4 [15, 16] and TG/HDL-C <2 [17] were considered desirable.

In order to evaluate the age dependency, both PCOS and controls were divided into two subgroups: younger and older than 30 years. Additionally, in order to evaluate the influence of hyperandrogenism, PCOS group was also divided into two subgroups according to FAI quintiles.

### 2.3. Statistical Analysis

Statistical analysis was performed using the Statistical Package for Social Science (version 13.0, SPSS Inc., Chicago, IL). The level of significance was set on 0.05. For comparison between groups, Student's *t*-test for two independent samples for parametric data and *χ* ^2^ test for nonparametric data and binomial variables were used. Results are presented as mean ± standard deviation (*x* ± SD) for continuous variables and as percentages for binary variables. Associations between different variables were determined using Spearman correlation coefficient with two-tailed test of significance. In order to analyze potential predictors of SBP and DBP, univariant linear regression analysis was performed with parameters that significantly correlated with SBP and DBP. All significant predictors form univariant regression analysis entered stepwise multiple linear regression analysis. In order to get the cut-off values of independent predictors for hypertension, we generated the receiver operating characteristic (ROC) curves separately in subgroups PCOS <30 years and PCOS ≥30 years. The areas under the curve (AUCs) are provided with standard error of mean (SEM) and 95% confidence intervals (95% CI). The ROC curves, a plot of the sensitivity (SEN) (true positive) versus 1-specificity (SP) (false positive) for each predictor tested, determine the ability of a screening measure for correctly identifying individuals based on their classification by a reference test. The values for each AUC can be between 0 and 1, with a value of 0.5, indicating that the diagnostic test is no better than chance. We considered that parameter has an accurate diagnostic sensitivity when AUC value was >0.75. We defined the best cut-off value as the value with the highest proportion of positives and negatives classified correctly by the test.

## 3. Results

### 3.1. Clinical Characteristics of Women with PCOS and Controls

Clinical characteristics and respective biochemical analyses for the whole group of PCOS women and controls are presented in Table 1. Groups were comparable by age and BMI, but PCOS had higher degree of centripetal obesity (WHR, *P* = 0.03). SBP and DBP, lipid parameters, fasting glucose, insulin, and HOMA index did not differ between groups. As expected, women with PCOS had significantly higher levels of testosterone and FAI.

There were no differences between PCOS and controls in the prevalence of hypertension (30% versus 20%, *P* = 0.192), increased TC/HDL-C ratio (≥4) (38% versus 28%, *P* = 0.225), or TG/HDL-C (9% versus 8%, *P* = 0.552). There was no significant difference in CIMT between PCOS and controls on either ACC (0.54 ± 0.63 versus 0.47 ± 0.07, resp.) or ACI (0.53 ± 0.63 versus 0.47 ± 0.07, resp.).

### 3.2. Effect of Aging on Lipid Parameters

For the more accurate analyses of the cardiovascular risk (CVR) factors related to age, all subjects were divided using age cut-off of 30 years and the differences are presented in Table 2. Except the expected difference in levels of androgens, PCOS and controls younger than 30 years did not differ in any other metabolic or cardiovascular indices. PCOS older than 30 years had higher BMI, WHR, SBP, DBP, all lipids and their ratios, and Apo-B than PCOS younger than 30 years. Significant difference remained for SBP, DBP, TG, insulin, and FAI after the adjustment for BMI. PCOS older than 30 years had higher WHR, SBP, DBP, TG, and TG/HDL-C than respective control subgroup.

Prevalence of hypertension was not different between PCOS and controls younger than 30 years (20% versus 22%, *P* = 0.801) while, in the older subgroups, PCOS had significantly higher prevalence of hypertension than controls (61% versus 17%, *P* = 0.003). In comparison to PCOS younger than 30 years, prevalence of hypertension was higher in PCOS in the fourth decade (*P* < 0.001), even after adjustment for BMI (*P* = 0.005).

Prevalence of TC/HDL-C ≥4 did not differ between PCOS and controls younger than 30 years (27% versus 25%, *P* = 0.807) while in the older subgroups it was more prevalent in PCOS in comparison to controls (71% versus 33%, *P* = 0.016). Prevalence of TC/HDL-C ≥4 was higher in PCOS in the fourth decade in comparison to those younger than 30 years (*P* < 0.001), even after adjustment for BMI (*P* = 0.025). There was no difference in the prevalence of TG/HDL-C ≥2 between PCOS and controls younger than 30 years (*P* = 0.402) and older than 30 years (*P* = 0.214). In comparison to PCOS younger than 30 years, prevalence of TG/HDL-C ≥2 was higher in PCOS in the fourth decade (20% versus 5%, *P* = 0.034) but remained insignificant after adjustment for BMI (*P* = 0.693).

PCOS and controls younger than 30 years and older than 30 years had similar CIMT on ACC (*P* = 0.197 and 0.142, resp.) and ACI (*P* = 0.201 and 0.103, resp.). PCOS ≥30 years had higher CIMT on ACC in comparison to PCOS <30 years (0.47 ± 0.08 versus 0.44 ± 0.06, *P* = 0.043), even after adjustment for BMI (*P* = 0.036), while there was no difference on ACI (*P* = 0.299). Controls ≥30 years had higher CIMT on ACC in comparison to controls <30 years (0.50 ± 0.06 versus 0.46 ± 0.05, *P* = 0.009), while there was no difference on ACI (*P* = 0.060).

### 3.3. Clinical and Biochemical Characteristics Related to the Level of FAI

For the assessment of possible influence of androgens on cardiovascular risk factors and outcomes, PCOS group was analyzed related to the quintiles of FAI. Subgroup with the highest quintile of FAI (≥11) was compared to the joint subgroup consisting of subjects with FAI <11. The PCOS subgroups did not differ in age, but women with the highest FAI quintile were more obese in comparison to the women with lower FAI (29.04 ± 7.04 versus 23.36 ± 5.34 kg/m^2^, *P* < 0.001). Although PCOS subgroup with the highest FAI quintile in comparison to the joint PCOS subgroup with lower FAI had higher SBP, DBP, TG, and lipid ratios, the only significant differences that remained after adjustment for BMI were for the ratios of TC/HDL (4.58 ± 1.28 versus 3.46 ± 0.99, *P* = 0.045) and TG/HDL (1.47 ± 0.93 versus 0.81 ± 0.65, *P* = 0.034). Prevalence of hypertension was not different among the subgroups of FAI (39% versus 27%, *P* = 0.230). There were no differences in CIMT related to FAI subgroups on either ACC or ACI, even after BMI adjustment (*P* = 0.076 and *P* = 0.100, resp.).

### 3.4. Correlations of Clinical and Biochemical Parameters

In PCOS subjects, SBP significantly correlated with age (rho = 0.317, *P* < 0.001), BMI (rho = 0.375, *P* < 0.001), WHR (rho = 0.322, *P* < 0.001), TG (rho = 0.236, *P* = 0.011), TG/HDL-C (rho = 0.236, *P* = 0.010), TC/HDL-C (rho = 0.265, *P* = 0.004), and Apo-B (rho = 0.302, *P* < 0.001). DBP significantly correlated with age (rho = 0.362, *P* < 0.001), BMI (rho = 0.383, *P* < 0.001), WHR (rho = 0.336, *P* < 0.001), TG (rho = 0.284, *P* = 0.002), TG/HDL-C (rho = 0.298, *P* = 0.001), TC/HDL-C (rho = 0.309, *P* = 0.001), Apo-A1 (rho = −0.227, *P* < 0.017), Apo-B (rho = 0.328, *P* < 0.001), and FAI (rho = 0.215, *P* = 0.021). TC/HDL-C significantly correlated with WHR (rho = 0.549, *P* < 0.001), BMI (rho = 0.552, *P* < 0.001), HOMA (rho = 0.193, *P* = 0.040), and FAI (rho = 0.369, *P* < 0.001). TG/HDL-C significantly correlated with WHR (rho = 0.524, *P* < 0.001), BMI (rho = 0.569, *P* < 0.001), HOMA (rho = 0.311, *P* = 0.001), and FAI (rho = 0.432, *P* < 0.001). CIMT did not correlate with either lipid parameters or their specific indices in PCOS group.

In control subjects, SBP significantly correlated with total testosterone (rho = 0.359, *P* = 0.010), DHEAS (rho = 0.288, *P* = 0.045), and androstenedione (rho = 0.435, *P* = 0.003), while DBP significantly correlated with WHR (rho = 0.331, *P* = 0.019), total testosterone (rho = 0.338, *P* = 0.016), FAI (0.346, *P* = 0.021), and androstenedione (rho = 0.435, *P* = 0.003). TC/HDL-C significantly correlated with WHR (rho = 0.418, *P* < 0.001), BMI (rho = 0.555, *P* < 0.001), HOMA (rho = 0.424, *P* = 0.001), and FAI (rho = 0.519, *P* < 0.001), while TG/HDL-C correlated with WHR (rho = 0.329, *P* = 0.020), BMI (rho = 0.492, *P* < 0.001), and FAI (rho = 0.518, *P* < 0.001).

In the subgroup of PCOS <30 years, SBP and DBP significantly correlated with BMI (rho = 0.265, *P* = 0.013 and rho = 0.309, *P* = 0.003, resp.) and DHEAS (rho = 0.263, *P* = 0.014 and rho = 0.266, *P* = 0.013, resp.). WHR significantly correlated with TC (rho = 0.329, *P* = 0.02), HDL (rho = −0.271, *P* = 0.011), LDL (rho = 0.357, *P* = 0.001), TG (rho = 0.416, *P* < 0.001), TC/HDL (rho = 0.451, *P* < 0.001), TG/HDL (rho = 0.398, *P* < 0.001), Apo-B (rho = 0.439, *P* < 0.001), insulin (rho = 0.399, *P* < 0.001), HOMA (rho = 0.398, *P* < 0.001), and SHBG (rho = −0.330, *P* = 0.002). In PCOS ≥30 years SBP and DBP significantly correlated with WHR (rho = 0.621, *P* < 0.001 and rho = 0.472, *P* = 0.011, resp.), TG (rho = 0.467, *P* = 0.012 and rho = 0.595, *P* = 0.001, resp.), Apo-B (rho = 0.473, *P* = 0.015 and rho = 0.393, *P* = 0.047, resp.), and TG/HDL-C (rho = 0.473, *P* = 0.011 and rho = 0.589, *P* = 0.001, resp.). Besides the above mentioned correlations with SBP and DBP, WHR significantly correlated also with TG (rho = 0.521, *P* = 0.004), TG/HDL (rho = 0.545, *P* = 0.003), Apo-B (rho = 0.438, *P* = 0.025), insulin (rho = 0.510, *P* = 0.006), HOMA (rho = 0.502, *P* = 0.008), and SHBG (rho = −0.532, *P* = 0.004).

In PCOS with the highest FAI, TG/HDL-C significantly correlated with WHR (rho = 0.471, *P* = 0.006) and BMI (rho = 0.585, *P* < 0.001). SBP and DBP significantly correlated with WHR (rho = 0.396, *P* = 0.023 and rho = 0.384, *P* = 0.027, resp.) and BMI (rho = 0.506, *P* = 0.003 and rho = 0.423, *P* = 0.014, resp.). In PCOS with lower FAI, TG/HDL-C significantly correlated with WHR (rho = 0.417, *P* < 0.001) and BMI (rho = 0.409, *P* < 0.001). SBP and DBP significantly correlated with WHR (rho = 0.255, *P* = 0.020 and rho = 0.264, *P* = 0.016, resp.) and BMI (rho = 0.262, *P* = 0.017 and rho = 0.320, *P* = 0.003, resp.).

### 3.5. Linear Regression Analysis

In order to determine significant predictors for SBP and DBP in the PCOS group, we performed uni- and multivariate linear regression analyses with SBP and DBP as dependent variables, separately in PCOS <30 years and PCOS ≥30 years. Independent variables included in analyses were only those that significantly correlated with SBP and DBP in both PCOS subgroups. Accordingly, dependent predictors for SBP and DBP in PCOS <30 years were BMI and DHEAS, and both of those remained significant predictors of SBP in multiple regression analysis (Table 3). In PCOS ≥30 years dependent variables for both SBP and DBP were WHR, Apo-B, and TG/HDL-C. In multiple linear regression analysis, significant predictors of SBP remained TG/HDL-C and WHR, while the only significant predictor for DBP was TG/HDL-C (Table 3).

### 3.6. ROC Curve Analysis

We have performed the ROC curve analysis for various predictors of hypertension in PCOS group.

In subgroup PCOS <30 years, BMI and DHEAS were considered. However, the resulting values BMI [AUC: 0.676 ± 0.065 (0.549–0.803), *P* = 0.022] and DHEAS [AUC: 0.636 ± 0.083 (0.474–0.798), *P* = 0.077] appeared to be insignificant according to our presumptions (see methodology), so the cut-off values for those indices could not be formed.

In subgroup PCOS ≥30 years, TG/HDL-C exhibited high diagnostic accuracy for prediction of hypertension [AUC: 0.81 ± 0.083 (0.644–0.971), *P* = 0.007]. Cut-off value 1.10 had SEN: 82.4% and SP: 72.7% for prediction of hypertension in PCOS ≥30 years. The obtained cut-off value had higher positive predictive value (PPV 82%) than negative predictive value (NPV 73%) (*P* = 0.006). Yet, in the general PCOS population, this cut-off value produced higher negative (NPV 76%) than positive predictive value (PPV 51%) (*P* = 0.004).

## 4. Discussion

In this study, we obtained significantly higher values of SBP and DBP as well as the prevalence of hypertension in our population of PCOS women over 30 years of age in comparison to younger PCOS women and respective controls. The same type of the age-related change towards higher values in PCOS women over 30 years was obtained for triglycerides. Moreover, women with PCOS and the most pronounced hyperandrogenemia, mirrored through FAI, had significantly higher proatherogenic lipid indexes as TG/HDL and TC/HDL, thus implicating the role of androgens in such metabolic derangements. These results were maintained after adjustment for the BMI of the investigated PCOS women. According to the literature data, this is the first study to show an aggravation of arterial blood pressure and lipid indices in the PCOS age group over 30 years after adjustment for BMI as the most disputable confounding factor in related studies.

The assessment of arterial blood pressure represents a valuable clinical surrogate maker for coexisting CVD. The prevalence of hypertension varies in the studied PCOS populations and their clinical phenotypes [18, 19]. One explanation could be the impact of ethnicity. However, it is still unclear whether PCOS women have a higher prevalence of hypertension in comparison to reproductive healthy women or not, because it is not usually controlled for obesity in the majority of studies [20]. In our study, hypertension was more prevalent within the older PCOS group even when adjusted for BMI.

It has been shown that women with PCOS are frequently developing centripetal obesity [21]. Particularly, the abdominal phenotype of obesity is a clinical predictor of metabolic abnormalities that can be detected in the early stages of PCOS and could even precede its development [3]. Our results showed that women with PCOS from the fourth decade of life are more prone to develop centripetal obesity thus representing a possible starting point for the development of other metabolic disorders. This observation is in line with the obtained significant positive correlations between WHR and reliable clinical markers of CVD (TG/HDL ratio, SBP, and DBP) in our PCOS women. The above obtained associations indicate the complex causative relationship between abdominal obesity, the consequent development of insulin resistance, and even more type 2 diabetes (T2D) that seems to be intrinsic to PCOS and their BMI increase [22, 23]. Obesity and insulin resistance predispose PCOS women to blood pressure dysregulation, thus making obesity a major link between PCOS and hypertension [24]. Moreover, hypertension was more prevalent within our PCOS women from the fourth decade of life in comparison to both age-comparable controls and younger PCOS, even after adjustment for BMI. However, we did not confirm aggravation of insulin resistance in PCOS in comparison to controls, although both groups were markedly insulin resistant. A possible explanation could lie in the method used for the evaluation of insulin resistance, thus lacking the precision to detect a difference [25].

The total cholesterol/HDL-cholesterol ratio was proved to be a relevant parameter for the assessment of the increased risk for CHD [16]. Although there was no age-related difference in this lipid index between our PCOS women and the controls, the prevalence of the TC/HDL-C ratio ≥4 was higher in older PCOS in comparison to both age-comparable controls and younger PCOS. As TC/HDL-C ratio ≥4 is independently associated with an increased prevalence of significant obstructive coronary artery disease [16], our results implicate higher CVR in PCOS with aging. Although we found a significant correlation between the TC/HDL-C ratio and both SBP and DBP, this index was not proved to be a relevant predictor of hypertension in our PCOS population. The TG/HDL-C ratio represents a reliable indirect indicator of the size of the LDL particles [26] and was proved to be good indicator for identifying subjects with insulin resistance and a risk for the development of T2D and CVD in presumably healthy individuals [27]. Our PCOS women older than 30 years had a significantly higher TG/HDL-C ratio than the controls. Moreover, this lipid index was confirmed as an independent predictor of both SBP and DBP in PCOS in the fourth decade of life with a high diagnostic accuracy for the diagnosing of hypertension in the whole PCOS group irrespective of age. It could be speculated that the proatherogenic potential could be translated even from the adolescent period [26] to the fourth and later decades of life, as the TG/HDL ratio reached a critical value in our PCOS women older than 30 years.

We need to emphasize that in defining hypertension we used the cut-off for SBP ≥130 mmHg and DBP ≥85 mmHg. These values are present in all the current criteria for the MetS that was shown to be prevalent in women with PCOS [28]. In comparison to other studies, our TG/HDL-C ratio cut-off value (1.10) for the prediction of hypertension in PCOS was slightly lower than that previously proposed as the most reliable in the discrimination of the atherogenic LDL phenotype and the identification of women with an accentuated cardiometabolic risk and T2D (1.33 and 2.0, resp.) [17, 26]. The difference in our cut-off value could be explained by the younger age of our study population. The proposed TG/HDL-C cut-off could be an early identification for PCOS with a risk of hypertension and first clinical indicator for clinicians to pursue more on the prevention strategies.

The effect of androgens on the blood pressure in women with PCOS is still a controversial issue. It is assumed that 60–80% of PCOS women have hyperandrogenemia [29] and that the cardiometabolic risk is prevalent in hyperandrogenic PCOS phenotypes [30]. Several studies have shown that a decreased SHBG and the consequent higher free testosterone positively correlated with triglycerides and insulin levels and negatively with HDL-C in both premenopausal women with PCOS and those after menopause [31, 32]. In our study, women with PCOS and the most pronounced hyperandrogenemia, mirrored through the FAI, had significantly higher proatherogenic lipid indexes as TG/HDL and TC/HDL, thus implicating the role of androgens in such metabolic derangements. These results were maintained after adjustment for the BMI of the investigated PCOS women. It was proposed that androgens increase the arterial blood pressure in women with PCOS by altering the components of the renin-angiotensin system. Namely, an increased expression of renin and angiotensin in the granulosa and theca cells in women with PCOS has been immunohistochemically proven [33]. On the other hand, it was presumed that androgen excess favours abdominal adiposity from an early age and consequently facilitates insulin resistance [34]. The existence of an aberrant morphology and function of adipose tissue in women with PCOS is associated with hypoxia-induced pathways leading to low-grade inflammation and the consequent production of cytokines and chemokines, as well as decreased production of adiponectin. A chronic low-grade inflammatory state has been associated with the development of local and systemic insulin resistance and, probably through this mechanism, with T2D and CVR factors [35]. It is suggested that androgens could represent mediators to these processes by stimulating the differentiation of preadipocytes into mature adipocytes and influencing the biochemical pathways of lipid and carbohydrate metabolism, as well as oxidative stress [36].

Besides arterial blood pressure, CIMT represents a vascular surrogate marker with the potential for the prediction of CVD in women with PCOS. It was suggested that an increased CIMT is prevalent in young women with PCOS [37]. However, some investigations either did not confirm any differences in CIMT in younger PCOS women [38, 39] or reported higher CIMT only in PCOS women over the age of 45 in comparison to the controls [38]. Our results are in line with this observation. Even more, our group of women is among the largest investigated populations of PCOS, thus giving more relevance to the lack of difference in CIMT between PCOS women and controls and an absence of any causative relation to other investigated CVR factors. Hence, it could be supposed that not every CVR in young women with PCOS is translated into the CVD or it is not yet apparent at this early stage of the condition [39]. Whether the cardiovascular risk translates into increased cardiovascular morbidity and mortality has not yet been fully elucidated as appropriate long-term prospective studies are lacking.

CVD risk assessment in women with PCOS is recommended at any age for blood pressure, glucose, lipid profile (cholesterol, triglycerides, HDL, LDL, and non-HDL cholesterol), waist circumference, physical activity, nutrition, and smoking. Moreover, periodic reassessment for CVD risk is recommended as CVD risk increases with age [40]. It is recognized that the prevention of future adverse cardiovascular effects is needed in women with PCOS. The primary prevention of CVD involves lifestyle modification including weight reduction using a low saturated fat diet and an individualized exercise program. This is recommended as first-line therapy for all women with PCOS and particularly for those with serum LDL-C levels >160 mg/dL (>4.14 mmol/L) and/or non-HDL-C levels >190 mg/dL (>4.92 mmol/L) when additional therapy with cholesterol-lowering drug therapy (statins) should be considered [41]. Recent meta-analysis of the randomized clinical trials with simvastatin (20 mg/day) and atorvastatin (20–40 mg/day) in women with PCOS showed their effectiveness at reducing LDL-C, total cholesterol, triglycerides, and testosterone levels [42]. At the initiation of drug therapy, monitoring of lipid levels should follow after 6 weeks [41].

## 5. Conclusion

We observed that women with PCOS possess the potential for early atherosclerosis which can be detected through elevated and deranged predictors of atherosclerosis, namely, lipids and changes in blood pressure. It seems that the potential for subclinical CVD becomes apparent from the fourth decade of life, irrespective of their BMI. A more proper assessment of the clinical phenotypes and use of specific metabolic indicators could be a valuable tool for the evaluation of cardiovascular potential and outcomes in future randomized studies on women with PCOS.

## Figures and Tables

**Table 1:** Clinical and biochemical parameters in women with PCOS and controls.

Parameter	PCOS (*n* = 100)	Controls (*n* = 50)	*P* value
Age (years)	26.32 ± 5.26	27.96 ± 5.66	0.082
BMI (kg/m^2^)	24.98 ± 6.38	24.66 ± 6.7	0.781
WHR	0.84 ± 0.11	0.80 ± 0.07	**0.033**
SBP (mmHg)	119.53 ± 11.28	117.60 ± 11.83	0.333
DBP (mmHg)	77.39 ± 9.47	75.90 ± 9.35	0.362
TC (mmol/L)	5.07 ± 1.01	4.98 ± 1.04	0.619
HDL-C (mmol/L)	1.36 ± 0.33	1.37 ± 0.29	0.857
LDL-C (mmol/L)	3.16 ± 0.87	3.12 ± 0.93	0.782
TG (mmol/L)	1.20 ± 0.74	1.07 ± 0.71	0.305
Glucose (mmol/L)	4.69 ± 0.51	4.61 ± 0.42	0.329
Insulin (mU/L)	16.35 ± 9.62	16.18 ± 9.04	0.920
HOMA	3.46 ± 2.28	3.30 ± 1.85	0.678
Apo-A1 (g/L)	1.59 ± 0.31	1.63 ± 0.32	0.434
Apo-B (g/L)	0.91 ± 0.29	0.87 ± 0.26	0.400
TC/HDL-C	3.90 ± 1.15	3.78 ± 1.21	0.542
TG/HDL-C	1.0 ± 0.79	0.87 ± 0.75	0.354
Testosterone (nmol/L)	2.66 ± 0.99	1.64 ± 0.60	**<0.001**
SHBG (nmol/L)	38.67 ± 23.18	55.66 ± 32.0	**<0.001**
FAI	10.15 ± 10.22	4.1 ± 2.8	**<0.001**
Androstenedione (ng/mL)	3.39 ± 1.36	2.14 ± 1.11	**<0.001**
DHEAS (nmol/L)	8.15 ± 4.01	5.33 ± 2.92	**<0.001**

BMI: body mass index; WHR: waist-to-hip ratio; SBP: systolic blood pressure; DBP: diastolic blood pressure; TC: total cholesterol; HDL-C: high-density lipoprotein; LDL-C: low-density lipoprotein; TG: triglycerides; HOMA: homeostatic model assessment; Apo-A1: apolipoprotein A1; Apo-B: apolipoprotein B; DHEAS: dehydroepiandrosterone sulfate; SHBG: sex-hormone-binding globulin; FAI: free androgen index.

**Table 2:** Clinical and biochemical parameters in women with PCOS and controls related to age.

Parameter	Age <30 years (*n* = 108)			Age ≥30 years (*n* = 42)			PCOS <30 years versus PCOS ≥30 years		Controls <30 years versus controls ≥30 years *P* value
	PCOS (*n* = 76)	Controls (*n* = 32)	*P* value	PCOS (*n* = 24)	Controls (*n* = 18)	*P* value	*P* value	*P* value adjusted for BMI	
BMI (kg/m^2^)	23.32 ± 4.94	24.10 ± 6.65	0.507	30.2 ± 7.6	25.69 ± 6.86	0.059	**<0.001**	/	0.437
WHR	0.82 ± 0.11	0.79 ± 0.07	0.209	0.89 ± 0.09	0.81 ± 0.07	**0.006**	**0.008**	0.558	0.442
SBP (mmHg)	116.97 ± 9.14	117.66 ± 11.84	0.744	127.68 ± 13.62	117.5 ± 12.16	**0.016**	**<0.001**	**0.003**	0.965
DBP (mmHg)	75.28 ± 8.17	75.31 ± 9.67	0.986	84.11 ± 10.31	76.94 ± 8.93	**0.023**	**<0.001**	**0.003**	0.559
TC (mmol/L)	4.94 ± 9.60	4.84 ± 0.89	0.597	5.46 ± 1.07	5.23 ± 1.24	0.523	**0.028**	0.235	0.204
HDL-C (mmol/L)	1.40 ± 0.34	1.36 ± 0.26	0.493	1.23 ± 0.27	1.4 ± 0.32	0.076	**0.028**	0.669	0.608
LDL-C (mmol/L)	3.07 ± 0.82	2.97 ± 0.69	0.555	3.49 ± 0.99	3.39 ± 1.23	0.770	**0.045**	0.294	0.128
TG (mmol/L)	1.04 ± 0.52	1.13 ± 0.82	0.604	1.71 ± 1.06	0.98 ± 0.45	**0.009**	**<0.001**	**0.050**	0.483
Glucose (mmol/L)	4.64 ± 0.47	4.62 ± 0.37	0.793	4.87 ± 0.61	4.61 ± 0.51	0.145	0.056	0.337	0.936
Insulin (mU/L)	15.83 ± 8.87	14.73 ± 7.55	0.545	17.96 ± 11.71	19.08 ± 11.16	0.765	0.348	**0.028**	0.117
HOMA	3.30 ± 1.99	3.0 ± 1.49	0.451	3.96 ± 3.02	3.89 ± 2.37	0.942	0.331	0.080	0.183
Apo-A1 (g/L)	1.61 ± 0.33	1.68 ± 0.36	0.329	1.51 ± 0.25	1.54 ± 0.22	0.706	0.202	0.790	0.145
Apo-B (g/L)	0.87 ± 0.29	0.82 ± 0.22	0.383	1.04 ± 0.27	0.95 ± 0.31	0.354	**0.014**	0.490	0.074
TC/HDL-C	3.69 ± 1.10	3.68 ± 0.99	0.947	4.57 ± 1.09	3.96 ± 1.54	0.140	**0.001**	0.329	0.435
TG/HDL-C	0.84 ± 0.59	0.93 ± 0.86	0.520	1.51 ± 1.10	0.77 ± 0.49	**0.012**	**<0.001**	0.137	0.470
Testosterone (nmol/L)	2.56 ± 0.95	1.70 ± 0.60	**<0.001**	2.99 ± 1.05	1.53 ± 0.61	**<0.001**	0.061	0.158	0.348
SHBG (nmol/L)	39.19 ± 20.94	53.18 ± 27.19	**0.012**	37.03 ± 29.63	60.06 ± 39.65	**0.037**	0.693	0.167	0.472
FAI	9.76 ± 10.95	4.20 ± 2.68	**<0.001**	11.39 ± 7.55	3.92 ± 3.07	**<0.001**	0.499	**0.043**	0.735
Androstenedione (ng/mL)	3.38 ± 1.27	2.27 ± 1.18	**<0.001**	3.44 ± 1.67	1.9 ± 0.95	**0.03**	0.862	0.964	0.302
DHEAS (nmol/L)	8.46 ± 4.18	6.03 ± 3.08	**0.004**	7.16 ± 3.29	4.12 ± 2.20	**0.002**	0.175	0.082	**0.025**

BMI: body mass index; WHR, waist-to-hip ratio; SBP: systolic blood pressure; DBP: diastolic blood pressure; TC; HDL-C: high-density lipoprotein; LDL-C: low-density lipoprotein; TG: triglycerides; HOMA: homeostatic model assessment; Apo-A1: apolipoprotein A1; Apo-B: apolipoprotein B; DHEAS: dehydroepiandrosterone sulfate; SHBG: sex-hormone-binding globulin; FAI: free androgen index.

**Table 3:** Independent predictors of systolic and diastolic blood pressure in PCOS related to age.

Predictors	Unstandardized coefficients *B*	95% confidence interval for *B*		*P* value
		Lower	Upper	
**PCOS <30 years**				
**SBP** (*R* ^2^ = 0.16)				
BMI	0.532	0.126	0.938	**0.011**
DHEAS	0.577	0.094	1.060	**0.020**
**DBP** (*R* ^2^ = 0.13)				
BMI	0.431	0.069	0.793	**0.020**
DHEAS	0.465	0.034	0.896	**0.035**
**PCOS ≥30 years**				
**SBP** (*R* ^2^ = 0.42)				
TG/HDL-C	4.908	0.307	9.509	**0.038**
WHR	57.050	2.058	112.043	**0.043**
**DBP** (*R* ^2^ = 0.47)				
TG/HDL-C	5.708	2.363	9.053	**0.002**

SBP: systolic blood pressure; BMI: body mass index; DHEAS: dehydroepiandrosterone sulfate; DBP: diastolic blood pressure; TG: triglycerides; HDL-C: high-density lipoprotein; WHR: waist-to-hip ratio.
